# Upregulation of circ‐IGF1R increased therapeutic effect of hypoxia‐pretreated ADSC‐derived extracellular vesicle by regulating miR‐503‐5p/HK2/VEGFA axis

**DOI:** 10.1111/jcmm.18471

**Published:** 2024-07-10

**Authors:** Rongfeng Shi, Pengfei Jia, Suming Zhao, Hongxin Yuan, Jiahai Shi, Hui Zhao

**Affiliations:** ^1^ Department of Interventional and Vascular Surgery Affiliated Hospital of Nantong University Nantong Jiangsu P.R. China; ^2^ Institute of Interventional and Vascular Therapy Affiliated Hospital of Nantong University Nantong Jiangsu P.R. China; ^3^ Department of Thoracic Surgery Affiliated Hospital of Nantong University Nantong Jiangsu P.R. China; ^4^ Nantong Key Laboratory of Translational Medicine in Cardiothoracic Diseases, Research Institution of Translational Medicine in Cardiothoracic Diseases Nantong Jiangsu P.R. China

**Keywords:** circ‐IGF1R, extracellular vesicle, HK2, miR‐503‐5p, VEGFA

## Abstract

Diabetes mellitus is a major cause of blindness and chronic ulcers in the working‐age population worldwide. Wound healing is deeply dependent on neovascularization to restore blood flow. Former research has found that differentially expressed circular RNAs (circRNAs) are associated with hyperglycaemia‐induced endothelial cell damage, and hypoxia‐pretreated adipose‐derived stem cells (ADSCs)‐extracellular vesicle (HEV) transplants have a more therapeutic effect to enhance wound healing in diabetic mice by delivery circRNA. The current investigation employed high‐throughput sequencing to identify circRNAs that are abnormally expressed between EV and HEV. The regulatory mechanism and predicted targets of one differentially expressed circRNA, circ‐IGF1R, were investigated utilizing bioinformatics analyses, luciferase reporter assays, angiogenic differentiation assays, flow cytometric apoptosis analysis and RT‐qPCR. Circ‐IGF1R expression increased in HEV, and downregulation of circ‐IGF1R suppressed and reversed the promotion effect of HEV on angiogenesis in ulcerated tissue. Bioinformatics analyses and luciferase reporter assays confirmed that miR‐503‐5p was the downstream target of circ‐IGF1R, and inhibiting miR‐503‐5p restored the promotion effect of HEV on angiogenesis after circ‐IGF1R silence. The study also found that miR‐503‐5p can interact with 3'‐UTR of both HK2 and VEGFA. Overexpression of HK2 or VEGFA restored the promotion effect of HExo on angiogenesis after circ‐IGF1R silence. Overexpression miR‐503‐5p or silence HK2/VEGFA reversed the protective effect of circ‐IGF1R to MLMECs angiogenic differentiation. Overexpression of circ‐IGF1R increased the protective effect of HEV on the promotion of wound healing in mice with diabetes. Circ‐IGF1R promotes HIF‐1α expression through miR‐503‐5p sponging. Our data demonstrate that circ‐IGF1R overexpression EVs from ADSCs suppress high glucose‐induced endothelial cell damage by regulating miR‐503‐5p/HK2/VEGFA axis.

## 1 INTRODUCTION

Acute and chronic skin injuries caused by burns, stress, diabetes and venous stasis have brought a huge burden to society.^1^, ^2^ Normal wound healing is one of the most complex biological processes. It requires the precise coordination of many kinds of cells and accurate coordination of various biological and molecular events.^3^ Despite significant investment in this area, progress has been limited, especially in treating chronic wounds.

Stem cell‐based therapies open a new door for tissue repair and have been widely studied in regeneration medicine.^4^ Adipose stem cells (ASCs) have been well documented to have therapeutic effects on skin wound healing, cardiac injury, immune disorders and other indications of ischaemia and tissue loss.^5^ Recent studies have shown that paracrine factors significantly promote the therapeutic effect of stem cells on tissue repair, and extracellular vesicles (EVs) may play an important role.^6^ Harnessing their regenerative potential could overcome many of the translation barriers to cell therapy.

Extracellular vesicles composed of exosomes and microvesicles are considered to be the mediators of intercellular communication, allowing the exchange of DNA, RNA, proteins and lipids between cells.^7^ Exosomes are small vesicles with a diameter of 50–200 nm, whose secretion requires multi‐vesiculated endosomes to fuse with the plasma membrane.^8^, ^9^ In recent years, the role of EVs from different cell types in tissue repair has been extensively studied. The release of EVs is now considered to be one of the mediators of the therapeutic activities of MSCs.^10^, ^11^ Our previous research confirms that exosomes derived from mmu_circ_0000250‐modified adipose‐derived mesenchymal stem cells promote wound healing in diabetic mice by inducing miR‐128‐3p/SIRT1‐mediated autophagy.^12^ However, molecular mechanisms remain mysterious.

In the current investigation, we found that EVs from hypoxia adipose‐derived stem cells (ADSC‐HEV) are more effective in promoting wound healing compared with ADSC‐Exo in mice with diabetes. Circ‐IGF1R functioned in ADSC‐HEV‐mediated wound repair in mice with diabetes. However, the regulatory mechanism of circ‐IGF1R is unclear. So, the aim of this study was to reveal the regulatory mechanism of circ‐IGF1R for wound repair and clarify whether circ‐IGF1R can increase the therapeutic effect of hypoxia‐pretreated ADSC‐derived EVs.

## 2 MATERIALS AND METHODS

### 2.1 Ethics statement

Animal Care and Use Committee in the Affiliated Hospital of Nantong University approved the investigation. We conducted postoperative animal care and surgical treatment interventions according to the National Institutes of Health Guide for Laboratory Animals Care and Use.

### 2.2 ADSC‐EV identifications

Technicians cultivated ADSCs that had normoxic conditions in 95% air with 20% O_2_ and 5% CO_2_. For hypoxic pretreatments, technicians cultivated ADSCs in hypoxic conditions with 93% N_2_, 5% CO_2_ and 2% O_2_. When getting 80%–90% confluence, technicians rinsed ADSCs using PBS, which we cultivated in EGM‐2MV media without FBS and supplemented with 1× serum replacement solution (PeproTech, NJ, USA) for another 2 days. We got conditioned medium from ADSCs, which we centrifuged at 300 × *g* for 10 min and 2 kg for another 10 min to erase apoptosis cells and cellular debris. Lastly, post‐centrifugation at 12 kg for 30 min, technicians made the procedures at 4°C. We defined EV protein content employing Pierce BCA Protein Assay Kit (Thermo Fisher Scientific, MA, USA). We stored ADSC‐EV at −80°C, which we used in the following experiments: transmission electron microscopy, NTA assay and Western blotting were applied to identify the selected EVs.

### 2.3 Strand‐specific RNA‐Seq library

Technicians obtained total RNA from ADSCs‐EV as well as ADSCs‐HEV employing TRIzol reagent (Invitrogen, CA, USA). We used 3 μg RNA from each specimen through VAHTS. Total RNA‐Seq (H/M/R) Library Prep kits from Illumina (Vazyme Biotech Co., Ltd, Nanjing, China) were used to erase ribosomal RNA. ncRNAs and mRNAs were retained. We processed RNA using 40 U RNase R (Epicentre) at 37°C for 3 h and made TRIzol purification. We prepared the RNA‐Seq library via KAPA‐stranded RNA‐Seq Library Prep kits (Roche, Basel, Switzerland), which were applied for high‐throughput sequencing (Illumina HiSeq 4000 at Aksomics, Inc., Shanghai, China).

### 2.4 Bioinformatics analysis

Predicted interactions between circRNA, miRNA and mRNA were identified using the StarBase database.

### 2.5 Cell culture and cell transfection

Our lab gained MLMECs in ScienCell, which were cultivated in DMEM (HyClone, UT, USA) along with 10% FBS with no Exos (Gibco, CA, USA). Technicians maintained cells in a humidified incubator at 37°C. We made a circ‐IGF1R overexpression vector by putting circ‐IGF1R cDNA into pcDNA3.1 vector. Our team synthesized miR‐503‐5p mimics and HK2/VEGFA siRNA via Genepharma (Suzhou, China). Technicians transfected cells via Lipofectamine 3000 (Invitrogen) according to protocols.

### 2.6 RNA isolation and RT‐qPCR detection

We extracted total RNA employing TRIzol reagent (Invitrogen). We made cDNA synthesis employing TransScript all‐in‐one First‐Strand cDNA Synthesis SuperMix (TransGen Biotech, Beijing, China). Technicians made PCR using a PCR instrument (Bio‐Rad, CA, USA) with 2× Taq PCR Master Mix (Solarbio, Beijing, China) following the protocol. Our team computed fold alternations using 2^−Ct^ method. PCR primers were: circ‐IGF1R: forward 5′‐CCACAAATCGCTGCCAG‐3′, reverse 5′‐CGTTGCGGATGTCAATGC‐3′; miR‐503‐5p: forward 5′‐CGTAGCAGCGGGAACAGTT‐3′, reverse 5′‐AGTGCAGGGTCCGAGGTATT‐3′; NFE2L2: forward 5′‐CTTTAGTCAGCGACAGAAGGAC‐3′, reverse 5′‐AGGCATCTTGTTTGGGAATGTG‐3′; VEGFA: forward 5′‐CTGCCGTCCGATTGAGACC‐3′, reverse 5′‐CCCCTCCTTGTACCACTGTC‐3′; HK2: forward 5′‐TTCAGTCTCCGGCATAGCAAG‐3′, reverse 5′‐CATCTCCATGGCACTCTCTGG‐3′; U6: forward 5′‐TCGGCAGCACATATACTAAAAT‐3′, reverse 5′‐CGCTTCACGAATTTGCGTGTCAT‐3′; GAPDH forward 5′‐GTCAACGGATTTGGTCGTATTG‐3′, reverse 5′‐CCGTTCTCAGCCATGTAGTT‐3′.

### 2.7 Dual‐luciferase reporter assay

HK2/VEGFA gene 3′‐UTR and circ‐IGF1R, containing miR‐503‐5p binding sites, were amplified through PCR. Technicians put fragments to several cloning sites of the pMIR‐REPORT luciferase miRNA expression reporter vector (Ambion, Austin, USA). MLMECs having 0.1 μg luciferase reporter vectors were co‐transfected with wild‐type (WT) or mutant‐type (MUT) HK2/VEGFA or HK2/VEGFA 3′‐UTR and miR‐503‐5p mimic or miR‐control using Lipofectamine 3000 (Invitrogen, CA, USA). Our group calculated relative luciferase activity by firefly luminescence normalization to Renilla luminescence via a dual‐luciferase reporter assay system (Promega, WI, USA) following protocols 2 days post‐transfection.

### 2.8 Tubule formation assay

We tested neovascularization in fibrin matrices. We seeded serum‐starved MLMECs in an endothelial basal medium onto plates coated with Matrigel (BD Biosciences, NJ, USA) to incubate them. Our team captured and photographed tubular structures formed in Matrigel using phase‐contrast microscopy. Lengths regarding tubes newly constituted in 10 fields that were randomly selected were calculated.

### 2.9 Diabetes wound induction

Our team used C57BL male mice to induce diabetes via single intraperitoneal injection of streptozotocin (STZ). We validated diabetes by taking blood from the tail vein 3 days post‐STZ administration. We regarded mice with fasting blood glucose levels >250 mg/dL as diabetic and maintained them for 1 month for experiments that followed. Technicians anaesthetized mice via intramuscular injections and eliminated hair from the dorsal leg region to sterilize the area. The technician applied a sterile biopsy punch to make a 4‐mm full‐thickness excisional wound. Technicians allocated mice randomly to subcutaneous injection with 200 μg ADSC‐Exos in 100 μL of PBS. Technicians euthanized mice post‐15 days and harvested skin specimens.

### 2.10 Immunohistochemical data

We fixed tissue specimens to embed them in paraffin. Technicians cultured sections over the night using primary antibodies against CD31 at 4°C and with secondary antibodies (Abcam) for 1 h at 37°C. We stained sections employing 3,3‐diaminobenzidine to counterstain them via haematoxylin. We conducted TUNEL staining to examine sections.

### 2.11 Statistical analyses

Statisticians denoted continuous variables by means ± SD to conduct one‐way variance comparisons via GraphPad Prism (GraphPad, CA, USA). The *p* value of ≤0.05 inferred statistical significance.

## 3 RESULTS

### 3.1 Circ‐IGF1R plays an important role in hypoxia‐pretreated ADSC‐derived EV (HEV) mediated promotes wound healing in diabetic mice

**FIGURE 1:**
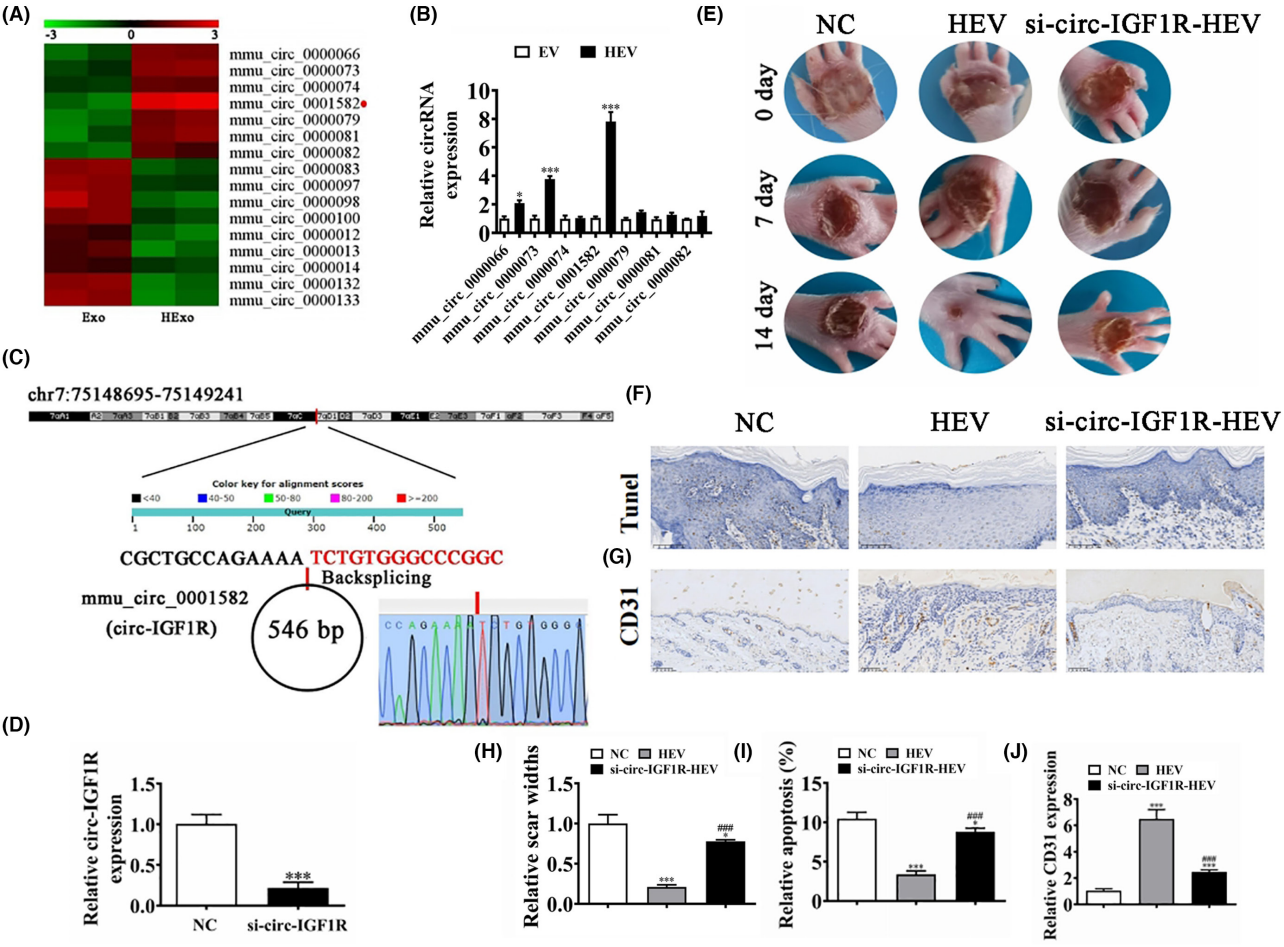
Circ‐IGF1R play an important role in hypoxia‐pretreated ADSC‐derived EV (HEV)‐mediated promotes wound healing in diabetic mice. (A) Clustered heat map of differentially expressed miRNAs depicting up‐ and down‐regulated miRNAs. (B) RT‐qPCR detection shows the different expression circRNAs. *p < 0.05, ***p < 0.001 versus EV. (C) Genomic loci of the *IGF1R* gene and mmu_circ_0001582. (D) RT‐qPCR detection shows the expression of circ‐IGF1R in HEV from ADSCs and circ‐IGF1R silence ADSCs. ***p < 0.001 versus NC. (E and H) Representative figures of wound healing procedures at various time points in various groups. The wound healing rate quantification. *p < 0.05, ***p < 0.001 versus NC. ^###^ *p* < 0.001 versus HEV. (F and I) Immunohistochemicals for Tunel staining show the apoptosis in skin tissue from the ulcer. *p < 0.05, ***p < 0.001 versus NC. ^###^ *p* < 0.001 versus HEV. (G and J) Immunohistochemicals for CD31 staining show the angiogenesis in skin tissue from the ulcer. ***p < 0.001 versus NC. ^###^ *p* < 0.001 versus HEV.

Our previous studies have confirmed that hypoxic ADSC‐derived EV (HEV) enhance wound healing in diabetic mice.^13^ In this study, we also found that circRNA were different expressions between HEV and EV, including mmu_circ_0000066, mmu_circ_0000073, mmu_circ_0000074, mmu_circ_0001582, mmu_circ_0000079, mmu_circ_0000081 and mmu_circ_0000082 (Figure 1A). RT‐qPCR detection shows that mmu_circ_0001582 expression was increased significantly in HEV compared with EV (Figure 1B). Using bioinformatics analysis (http://www.circbase.org/), we found that mmu_circ_0001582 was 546 bp and located within the *IGF1R* gene at chr7:75148695–75,149,241. Thus, mmu_circ_0001582 is referred to as circ‐*IGF1R* (Figure 1C). Generation sequencing also proved that circ‐IGF1R was the circRNA with back splicing. RT‐qPCR detection shows that circ‐IGF1R expression was decreased significantly after the silence of circ‐IGF1R in HEV (Figure 1D). We studied ADSC‐EV effects upon wound healing in full‐thickness cutaneous wounds of mice having STZ‐induced diabetes responding to subcutaneous injection regarding ADSC‐EV and ADSC‐HEV with equivalent volumes. We accelerated wound closure significantly using ADSC‐HEV compared with PBS controls. However, downregulation of circ‐IGF1R decreased the therapeutic effect of HEV to accelerated wound closure (Figure 1E,H). Immunohistochemicals for Tunel staining show that HEV treatment decreased apoptosis in ulcerated tissue. However, circ‐IGF1R silence decreased the protective effect of HExo (Figure 1F,I). CD31 staining shows that HEV treatment increased angiogenesis in ulcerated tissue. But circ‐IGF1R silence reversed the promotion effect of HEV on angiogenesis in ulcerated tissue (Figure 1G,J). A suggestion that circ‐IGF1R plays an important role in hypoxia‐pretreated ADSC‐derived EV (HEV) mediated promotes wound healing in diabetic mice.

### 3.2 MiR‐503‐5p was the downstream target of circ‐IGF1R

**FIGURE 2:**
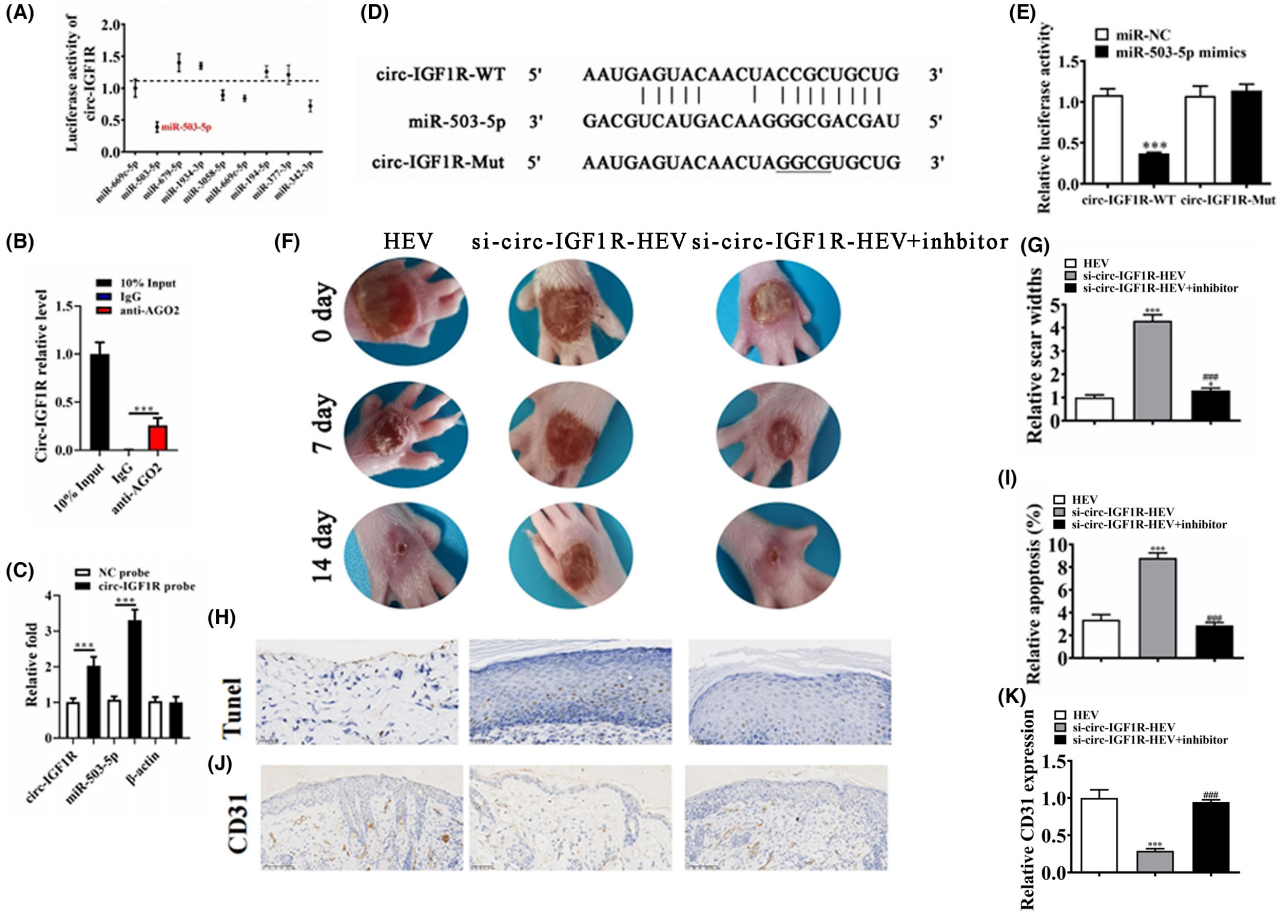
miR‐503‐5p was the downstream target of circ‐IGF1R. (A) Luciferase report analysis shows the downstream target of circ‐IGF1R. (B) AGO2 RNA‐binding protein immunoprecipitation. All data are shown as the mean ± SD. ***p < 0.001 compared to IgG. (C) circRNA pull‐down assay. **p < 0.01 compared to negative control (NC) probe. (D and E) Bioinformatics analysis and luciferase report analysis show the interactive relationship between miR‐503‐5p and circ‐IGF1R. ***p < 0.001. (F and G) Representative figures of wound‐healing procedures at various time points in various groups. The wound healing rate quantification. *p < 0.05, ***p < 0.001 versus HEV. ^###^ *p* < 0.001 versus si‐circ‐IGF1R‐HEV. (H and I) Immunohistochemicals for Tunel staining show the apoptosis in skin tissue from the ulcer. ***p < 0.001 versus HEV. ^###^ *p* < 0.001 versus si‐circ‐IGF1R‐HEV. (J and K) Immunohistochemicals for CD31 staining show the angiogenesis in skin tissue from the ulcer. ***p < 0.001 versus HEV. ^###^ *p* < 0.001 versus si‐circ‐IGF1R‐HEV.

Accumulation studies confirm that circRNA can regulate mRNA expression by sponges miRNA.^14^ In this study, we found that circ‐IGF1R can interact with miR‐669c‐5p, miR‐503‐5p, miR‐679‐5p, miR‐1934‐3p, miR‐3058‐5p, miR‐669c‐5p, miR‐194‐5p, miR‐377‐3p and miR‐342‐3p (Figure 2A). To validate this result, we conducted AGO2 RIP and found that endogenous circ‐IGF1R could be specifically pulled down by anti‐AGO2 antibody (Figure 2B). This suggests that circ‐IGF1R acts as a miRNA‐binding partner. Using a probe‐targeting circ‐IGF1R junction site, we found that circ‐IGF1R and miR‐503‐5p were significantly more abundant compared with the control (Figure 2C). Luciferase report analysis confirmed that only miR‐503‐5p can interact with circ‐IGF1R (Figure 2D,E). The in vivo experiment detection shows that inhibiting miR‐503‐5p restored the protective effect of HEV after circ‐IGF1R silence (Figure 2F,G). Immunohistochemicals for Tunel staining show that circ‐IGF1R silence decreased the protective effect of HEV. However, inhibiting miR‐503‐5p restored the protective effect of HEV after circ‐IGF1R silence (Figure 2H,I). CD31 staining shows that circ‐IGF1R silence reversed the promotion effect of HExo on angiogenesis in ulcerated tissue. However, inhibiting miR‐503‐5p restored the promotion effect of HExo on angiogenesis after circ‐IGF1R silence (Figure 2J,K). A suggestion was made that miR‐503‐5p was the downstream target of circ‐IGF1R.

### 3.3 Both HK2 and VEGFA were the downstream targets of miR‐503‐5p

**FIGURE 3:**
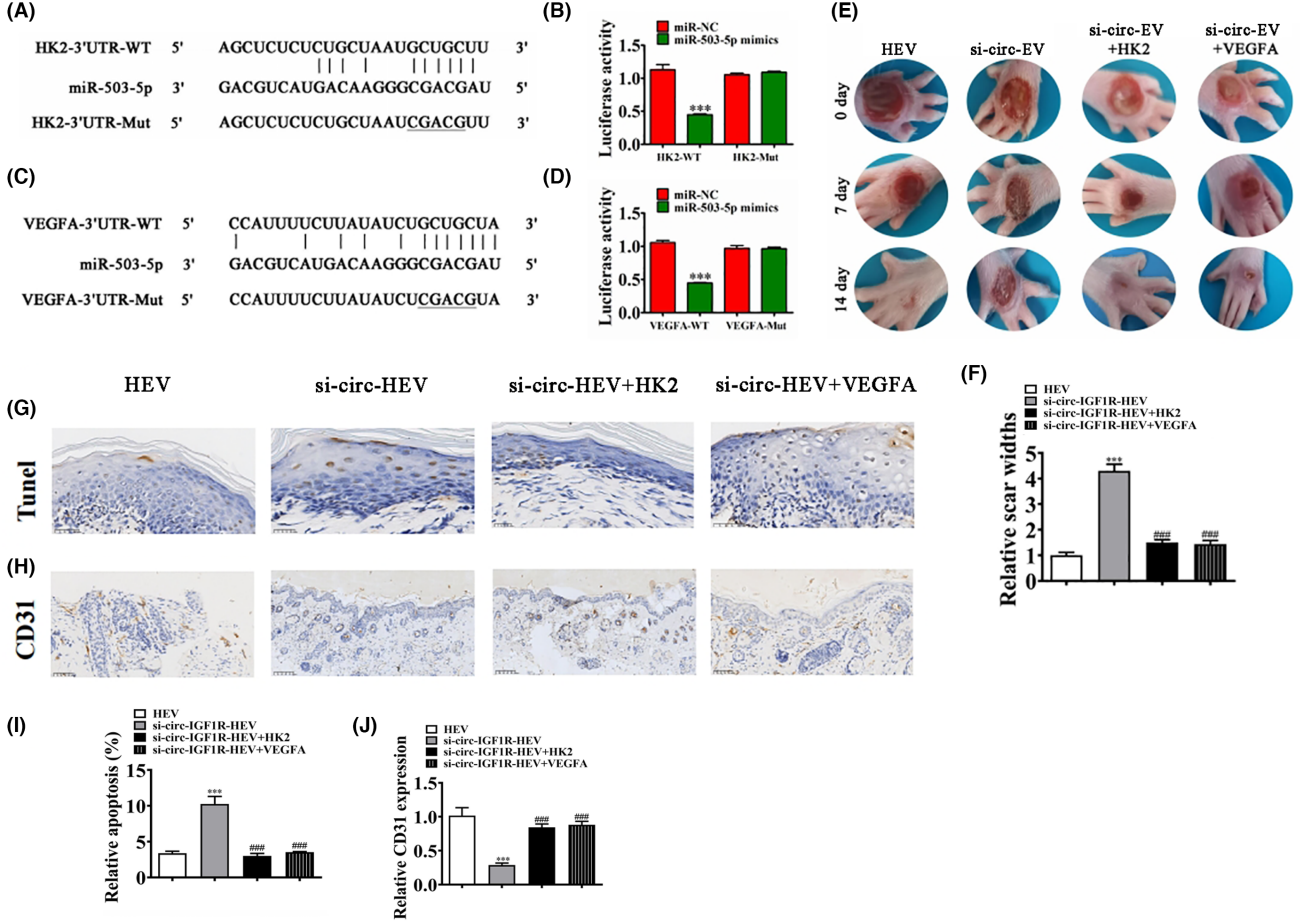
Both HK2 and VEGFA were the downstream target of miR‐503‐5p. (A and B) Bioinformatics analysis and luciferase report analysis show the interactive relationship between miR‐503‐5p and 3'‐UTR‐HK2. ***p < 0.001. (C and D) Bioinformatics analysis and luciferase report analysis show the interactive relationship between miR‐503‐5p and 3'‐UTR‐VEGFA. ***p < 0.001. (E and F) Representative figures of wound‐healing procedures at various time points in various groups. The wound healing rate quantification. ***p < 0.001 versus HEV. ^###^ *p* < 0.001 versus si‐circ‐IGF1R‐HEV. (G and H) Immunohistochemicals for Tunel staining show the apoptosis in skin tissue from the ulcer. ***p < 0.001 versus HEV. ^###^ *p* < 0.001 versus si‐circ‐IGF1R‐HEV. (I and J) Immunohistochemicals for CD31 staining show the angiogenesis in skin tissue from the ulcer. **p < 0.01, ***p < 0.001 versus HEV. ^###^ *p* < 0.001 versus si‐circ‐IGF1R‐HEV.

Luciferase report analysis confirmed that only miR‐503‐5p can interact with 3'‐UTR of both HK2 and VEGFA (Figure 3A–D). The detection of in vivo experiments shows that overexpression of HK2 or VEGFA restored the protective effect of HExo after circ‐IGF1R silence (Figure 3E,F). Immunohistochemicals for Tunel staining show that circ‐IGF1R silence decreased the protective effect of HEV. However, overexpression of HK2 or VEGFA restored the protective effect of HEV after circ‐IGF1R silence (Figure 3G,I). CD31 staining shows that circ‐IGF1R silence reversed the promotion effect of HExo on angiogenesis in ulcerated tissue. However, overexpression of HK2 or VEGFA restored the promotion effect of HEV on angiogenesis after circ‐IGF1R silence (Figure 3H,J). A suggestion was made that both HK2 and VEGFA were the downstream targets of miR‐503‐5p.

### 3.4 Overexpression miR‐503‐5p or silence HK2/VEGFA reversed the protective effect of circ‐IGF1R on vascular endothelial cell function under hyperglycaemic microenvironment

**FIGURE 4:**
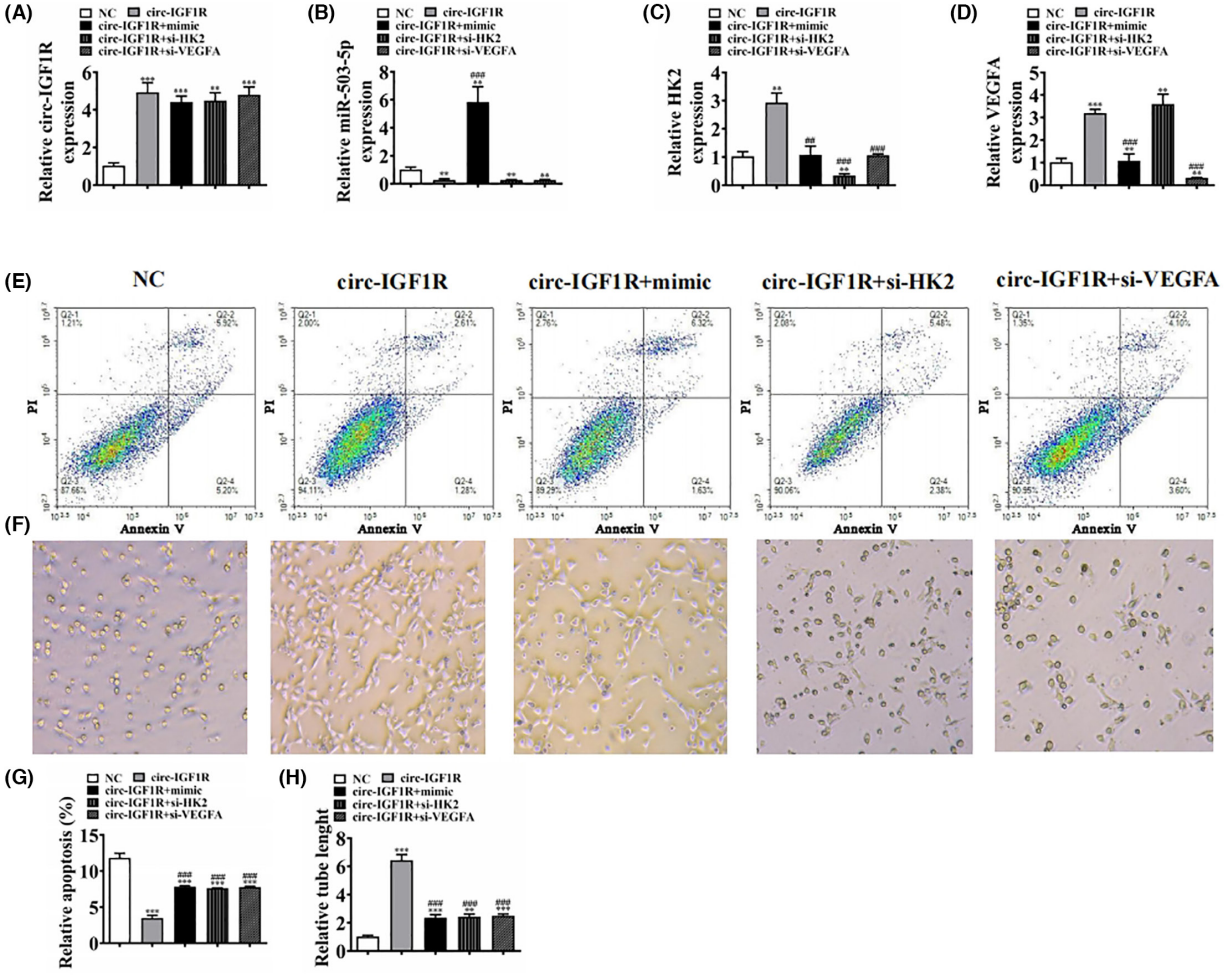
Overexpression miR‐503‐5p or silence HK2/VEGFA reversed the protective effect of circ‐IGF1R on vascular endothelial cell function under hyperglycemic microenvironment. (A–D) RT‐qPCR detection shows the expression of circ‐IGF1R, miR‐503‐5p, HK2 and VEGFA. **p < 0.01, ***p < 0.001 versus NC. ^###^ *p* < 0.001 versus circ‐IGF1R. (E and G) MLMECs apoptosis was assayed by flow cytometry after Annexin V‐FITC staining. ***p < 0.001 versus NC. ^###^ *p* < 0.001 versus circ‐IGF1R. (F and H) In vitro tube formation of MLMECs. The total branching was analysed. **p < 0.01, ***p < 0.001 versus NC. ^###^ *p* < 0.001 versus circ‐IGF1R. mimic, miR‐503‐5p mimic. NC, negative control.

In order to identify the interacted relationship among miR‐503‐5p, HK2, VEGFA and circ‐IGF1R, we constructed siRNA against HK2 and VEGFA and also constructed circ‐IGF1R overexpression vector and miR‐503‐5p mimic. We were then transfected into MLMECs. RT‐qPCR detection shows that circ‐IGF1R expression increased post‐transfection with circ‐IGF1R overexpression vector; at the same time, miR‐503‐5p mimic or HK2/VEGFA silence vector (si‐)HK2/VEGFA silence vector (si‐HK2/si‐VEGFA) treatments did not affect HK2 or VEGFA expressions (Figure 4A). Suggestion that HK2, VEGFA and miR‐503‐5p were the downstream target of circ‐IGF1R. RT‐qPCR unravelled that circ‐IGF1R overexpression decremented miR‐503‐5p expression. HK2 or VEGFA silencing had no effects upon circ‐IGF1R‐induced miR‐503‐5p downregulation (Figure 4B), suggesting that miR‐503‐5p was located at the downstream target of circ‐IGF1R. Data showcased that circ‐IGF1R overexpression increased both HK2 and VEGFA expression. miR‐503‐5p upregulation reversed the promotion effects of circ‐IGF1R on HK2 or VEGFA expression. Post‐HK2 or VEGFA silence, HK2 or VEGFA expression decreased significantly (Figure 4C,D). A suggestion was made that circ‐IGF1R promoted HK2 or VEGFA expression via miR‐503‐5p sponging.

MLMEC apoptosis was assayed by flow cytometry. The results show that overexpression of miR‐503‐5p or silence HK2/VEGFA reversed the protective effect of circ‐IGF1R on vascular endothelial cell survival under a hyperglycaemic microenvironment (Figure 4E,G). The data also revealed that overexpression miR‐503‐5p or silence HK2/VEGFA reversed the protective effect of circ‐IGF1R to MLMECs angiogenic differentiation (Figure 4F,H). Suggestion that overexpression miR‐503‐5p or silence HK2/VEGFA reversed the protective effect of circ‐IGF1R on vascular endothelial cell function under hyperglycemic microenvironment.

### 3.5 Circ‐IGF1R upregulation increased therapeutic effect regarding ADSC‐HEV on wound healing in mice with diabetes

**FIGURE 5:**
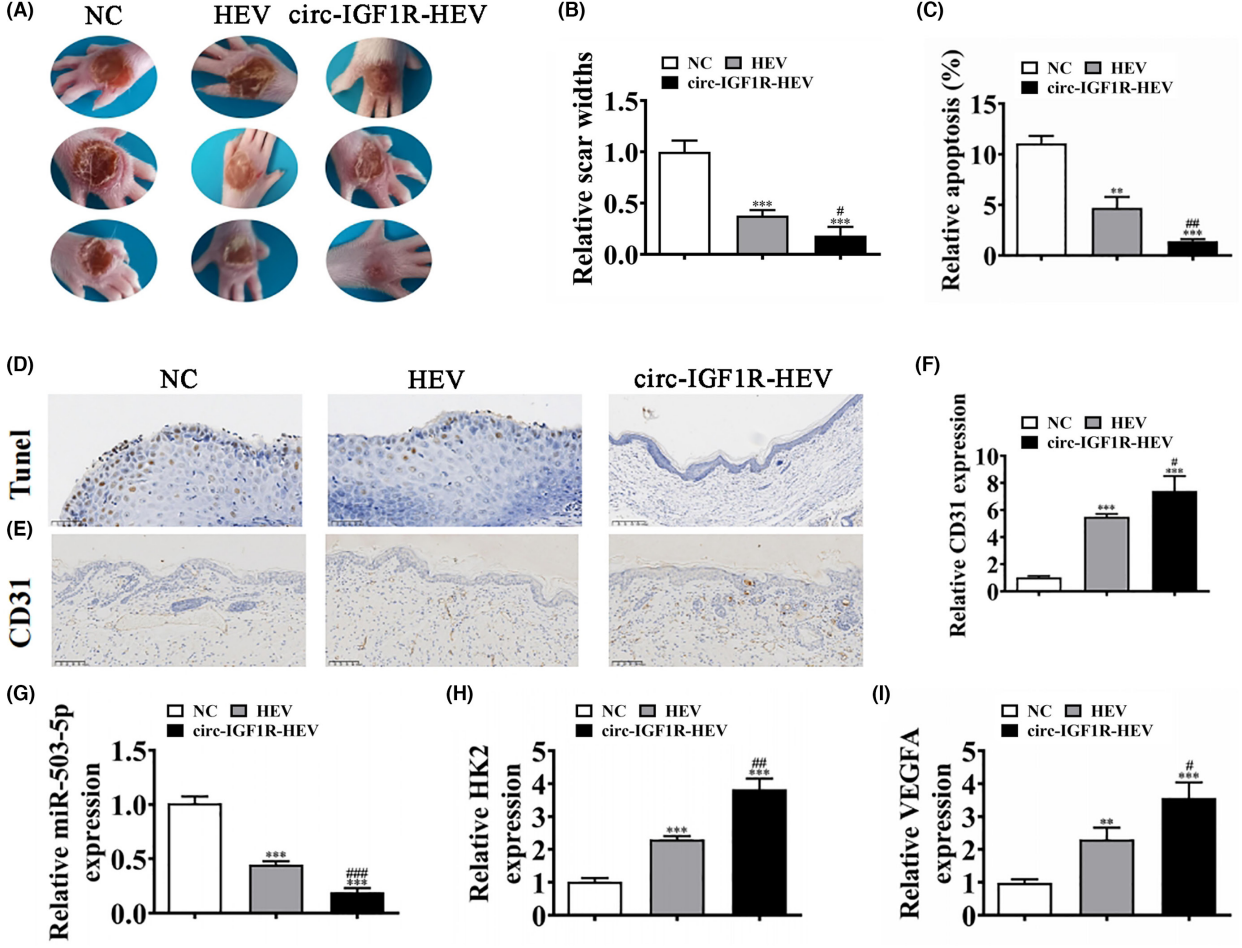
Circ‐IGF1R upregulation increased therapeutic effect of ADSC‐HEV on wound healing in diabetic mice. (A, B) Representative figures of wound‐healing procedures at various time points in various groups. The wound‐healing rate quantification. ***p < 0.001 versus NC. ^#^ *p* < 0.05 versus HEV. (C and D) Immunohistochemicals for Tunel staining show the apoptosis in skin tissue from the ulcer. **p < 0.01, ***p < 0.001 versus NC. ^##^ *p* < 0.01 versus HExo. (E and F) Immunohistochemicals for CD31 staining show the angiogenesis in skin tissue from the ulcer. ***p < 0.001 versus NC. ^#^ *p* < 0.05 versus HEV. (G–I) RT‐qPCR detection shows the expression of miR‐503‐5p, HK2 and VEGFA. **p < 0.01, ***p < 0.001 versus NC. ^#^ *p* < 0.05, ^##^ *p* < 0.01, ^###^ *p* < 0.001 versus HEV. ADSC‐HEV, hypoxia‐pretreated adipose‐derived stem cells‐extracellular vesicle; NC, negative control.

The in vivo experiment detection shows that overexpression of circ‐IGF1R increased the protective effect of HEV on promoting wound healing in mice with diabetes (Figure 5A,B). Immunohistochemicals for Tunel staining show that overexpression of circ‐IGF1R increased the protective effect of HEV in decreased apoptosis in ulcerated tissue in mice with diabetes (Figure 5C,D). CD31 staining shows that circ‐IGF1R overexpression promotes the angiogenesis of HEV in ulcerated tissue (Figure 5E,F). RT‐qPCR detection shows that overexpression of circ‐IGF1R decreased miR‐503‐5p expression more than HEV treatment ulcerated tissue in mice with diabetes (Figure 5G). However, overexpression of circ‐IGF1R increased HK2 and VEGFA expression, and HEV treatment ulcerated tissue in mice with diabetes (Figure 5H,I).

## 4 DISCUSSION

The refractory diabetic wound has remained a worldwide challenge as one of the major health problems. The impaired angiogenesis phase during diabetic wound healing partly contributes to the pathological process.^15^, ^16^ Our study found that exosomes derived from mmu_circ_0000250‐modified adipose‐derived mesenchymal stem cells promote wound healing in diabetic mice by inducing miR‐128‐3p/SIRT1‐mediated autophagy.^12^ Hypoxia adipose stem cell‐derived exosomes promote high‐quality healing of diabetic wounds involving activation of PI3K/Akt pathways.^17^ In this study, we also found that circ‐IGF1R plays an important role in hypoxia‐pretreated ADSC‐derived EV (HEV) mediated wound healing in diabetic mice. circ‐IGF1R was 546 bp and located within the *IGF1R* gene at chr7:75148695–75149241. Under high glucose stimulation, miR‐503 from M1 macrophage‐derived small EVs was taken by HUVEC‐targeted IGF1R in HUVECs and inhibited IGF1R expression. In HUVECs, miR‐503 inhibition improved hyperglycaemia‐caused HUVEC dysfunction, whereas IGF1R knockdown aggravated HUVEC dysfunction.^18^, ^19^ In this study, we also found that circ‐IGF1R plays an important role in hypoxia‐pretreated ADSC‐derived exosome (HEV) mediated wound healing in diabetic mice. Downregulation circ‐IGF1R reversed the promotion effect of HEV on angiogenesis in ulcerated tissue.

Bioinformatics and luciferase report analysis confirmed that miR‐503‐5p was the downstream target of circ‐IGF1R. Previous studies have confirmed that miR‐503‐5p were 6.21‐fold (*p* = 0.001) more highly expressed in diabetic foot patients than in healthy controls.^20^, ^21^ The study also found that miR‐503‐5p expression was increased under high glucose condition.^22^ Previous studies found that hsa_circ_0022742 expression in HUVECs was decreased by high glucose treatment, and overexpression of hsa_circ_0022742 suppressed high glucose‐induced endothelial dysfunction by target miR‐503‐5p.^23^ Our study also found that inhibiting miR‐503‐5p restored the promotion effect of HEV on angiogenesis after circ‐IGF1R silence. Suggestion that miR‐503‐5p was the target of circ‐IGF1R.

Further study also found that miR‐503‐5p can interact with 3'‐UTR of both HK2 and VEGFA. Accumulation studies confirmed that HIF‐1α stability and activation promote keratinocyte migration and wound repair.^24^ Moreover, HIF‐1α could bind with the promoter of hexokinase II (HK2) and promote its transcription.^25^ HK2 is important in regulating aerobic glycolysis, which promotes cell proliferation under stress microenvironment.^25^ Mechanistically, circHIPK3 promoted cell proliferation, migration and angiogenesis via downregulating miR‐20b‐5p to upregulate Nrf2 and VEGFA.^4^, ^26^ Inhibition of the HIF‐1α/VEGFA signalling using an inhibitor relieved the effect of cardamonin on cell viability, permeability and apoptosis in MLMECs under OGD/R.^27^ Our study found that HK2 or VEGFA silencing had no effects upon circ‐IGF1R‐induced miR‐503‐5p downregulation. Overexpression of HK2 or VEGFA restored the promotion effect of HEV on angiogenesis after circ‐IGF1R silence. Circ‐IGF1R upregulation increased the therapeutic effect of ADSC‐HEV on wound healing in mice with diabetes.

## 5 CONCLUSION

In conclusion, the current study demonstrated that EVs derived from hypoxia‐pretreated ADSCs enhance wound healing in diabetic mice via circ‐IGF1R delivery and induction, restoring angiogenesis function of vascular endothelial cells by promoted HK2/VEGFA via sponging miR‐503‐5p.

## AUTHOR CONTRIBUTIONS

**Rongfeng Shi:** Conceptualization (equal); formal analysis (equal); writing – review and editing (equal). **Pengfei Jia:** Conceptualization (equal); formal analysis (equal); writing – original draft (equal). **Suming Zhao:** Data curation (equal); formal analysis (equal). **Hongxin Yuan:** Data curation (equal); formal analysis (equal); visualization (equal); writing – original draft (equal). **Jiahai Shi:** Data curation (equal); funding acquisition (equal); investigation (equal); writing – review and editing (equal). **Hui Zhao:** Conceptualization (equal); data curation (equal); writing – original draft (equal); writing – review and editing (equal).

## FUNDING INFORMATION

This work was supported in part by the National Natural Science Foundation of China (Nos. 82102167, 82272093, 82370253), Jiangsu Provincial Research Hospital (YJXYY202204), Innovation Team Project of Affiliated Hospital of Nantong University (XNBHCX31773), Natural Science Foundation of Jiangsu Province of China (No. BK20180946), the Science Foundation of China Postdoctoral (No. 2019M651928).

## CONFLICT OF INTEREST STATEMENT

All the authors declare that they have no conflict of interest.

## Data Availability

The data sets used and/or analysed during the current study are available from the corresponding author upon reasonable request.
